# Evidence Supporting That RNA Polymerase II Catalyzes De Novo Transcription Using Potato Spindle Tuber Viroid Circular RNA Templates

**DOI:** 10.3390/v12040371

**Published:** 2020-03-27

**Authors:** Shachinthaka D. Dissanayaka Mudiyanselage, Ying Wang

**Affiliations:** Department of Biological Sciences, Mississippi State University, Starkville, MS 39762, USA; sdd292@msstate.edu

**Keywords:** RNA Polymerase II, potato spindle tuber viroid, RNA-templated transcription, de novo transcription, primed transcription

## Abstract

Transcription is a fundamental process that mediates the interplay between genetic information and phenotype. Emerging evidence indicates that RNA polymerase II (Pol II) can catalyze transcription using both DNA and RNA templates. It is well established that Pol II initiates de novo transcription on DNA templates. However, it is unclear whether Pol II performs de novo transcription or relies on primers for initiation (primed transcription) on RNA templates. Using potato spindle tuber viroid (PSTVd) as a model, we presented evidence showing that circular PSTVd templates are critical for the synthesis of longer-than-unit-length (−)-strand products, which supports the de novo transcription based on the asymmetric rolling circle model of PSTVd replication. We further showed that the crucial factor for primed transcription, transcription factor IIS (TFIIS), is dispensable for PSTVd replication in cells. Together, our data support the de novo transcription on PSTVd RNA templates catalyzed by Pol II. This result has significant implications in understanding the mechanism and machinery underlying Pol II-catalyzed transcription using other RNA templates.

## 1. Introduction

Transcription is a fundamental process that mediates the interplay between genetic information and phenotype and is thus vital for development, responses to environmental cues, and diseases [1,2,3,4]. As the first and indispensable step towards protein production, transcription is catalyzed by DNA-dependent RNA polymerases (DdRPs) that use DNA templates to synthesize RNA [5,6,7]. Since the first eukaryotic RNA polymerase identified in 1969 [8], the composition of the required machinery is well understood for DNA-dependent transcription through decades of research [6,9,10,11,12]. Each DdRP cooperatively associates with a group of factors to form the transcription machinery. Taking RNA polymerase II (Pol II) as an example, TFIID binds to DNA promoters and allures TFIIB, thereby constituting a platform to assemble the preinitiation complex (PIC) that includes TFIIA, TFIIB, TFIID, TFIIE, TFIIF, and TFIIH [13]. Subsequently, TFIIS and other factors together constitute the elongation complex [14].

It is known that DdRPs can use both DNA and RNA templates for transcription [15]. The RNA-dependent RNA polymerase (RdRP) activity of DdRPs is used by some RNA-based pathogens for replication, such as viroids and the human hepatitis delta virus (HDV) [16]. Viroids replicating in chloroplasts use the nucleus-encoded RNA polymerase (NEP) [17], while viroids replicating in the nucleus rely on Pol II [18,19,20,21,22]. Numerous lines of evidence support that Pol II catalyzes transcription/replication using HDV RNA templates [23,24,25], but there also exists evidence showing that Pol I and Pol III may participate in HDV replication [26,27,28,29,30,31,32]. The RdRP activity of DdRPs widely occurs in various organisms to regulate gene expression as well. For instance, under nutrient-deficient conditions, the bacterial DdRP interacts with the noncoding 6S RNA of *Escherichia coli* and stops transcribing DNA templates. In response to nutrient availability, the DdRP uses the 6S RNA template to transcribe a short RNA that leads to the de-sequestration of the DdRP. The free DdRP then binds to DNA promoters and initiates the synthesis of mRNAs [33]. Mammalian Pol II binds to a hairpin formed by the noncoding B2 RNA and extends the short strand by using the longer strand of the hairpin sequence as the template for transcription. This extension leads to the destabilization of the B2 RNA, representing a novel posttranscriptional mechanism to regulate RNA stability [34]. Therefore, the RdRP activity of DdRPs is emerging as a critical regulatory mechanism of gene expression across kingdoms, in addition to its function in pathogen infections.

DdRPs are well known for initiating de novo transcription on DNA templates [35]. However, it seems that DdRPs may have different modes when using RNA templates for transcription. Although the bacterial DdRP initiates de novo transcription when using the 6S RNA template [33], the evidence clearly shows that Pol II utilizes the fold-back 3′-end in the B2 RNA terminal loop as the primer to extend the B2 RNA [34]. This extension demonstrates that Pol II can perform the so-called primed transcription in eukaryotic cells, which is further supported by investigating the active Pol II structure on RNA templates [36]. It is also indicated that one general transcription factor, TFIIS, can promote the cleavage of RNA templates to generate an exposed 3′-end as RNA primers for transcription [36]. Whether Pol II exclusively performs primed transcription on RNA templates is a critical question in virology, because it directly relates to the replication of HDV and nuclear-replicating viroids. Whether HDV replication/transcription is primed remains to be clarified [31,32]. The primed transcription model may explain how Pol II catalyzes the synthesis of HDV mRNAs (HDag), but it is difficult to explain the rolling circle replication if the circular viral genome is cleaved [31,32]. Based on the primed transcription, it predicts the existence of the covalently linked templates and products, a chimeric intermediate remains to be identified [31,32]. Furthermore, the experimental systems supporting the primed transcription on RNA templates (B2 RNA and HDV) only generated very short RNA products [34,36,37,38,39], but the HDV concatemers are much longer in host cells. As for nuclear-replicating viroids, experimental evidence is needed to support the proposed de novo replication by Pol II.

We recently established an in vitro transcription (IVT) system for studying the Pol II-catalyzed replication of potato spindle tuber viroid (PSTVd), the type species of nuclear-replicating viroids [40]. We identified the eukaryotic transcription factor TFIIIA-7ZF as a critical factor facilitating Pol II-catalyzed transcription using the (+)-strand PSTVd RNA template [40,41]. We also mapped the TFIIIA-7ZF binding site [40], which is next to the transcription initiation site of (−)-strand intermediates [42] and is shown vital for Pol II binding [43] and PSTVd replication [44]. Therefore, this binding site likely acts as an RNA promoter. Furthermore, we observed the longer-than-unit-length products in the in vitro transcription assay [40] mimicking the process in cells [45]. Here, we improved the Pol II purification scheme for the IVT assay and directly verified the importance of circular templates for generating the longer-than-unit-length (−)-strand products, a critical intermediate in the rolling circle replication model [46,47,48]. We further showed that PSTVd replication was not affected in *tfiis* loss-of-function protoplasts, supporting the de novo replication of PSTVd by Pol II. Our observations have significant implications in understanding the molecular mechanism of Pol II-catalyzed transcription on RNA templates.

## 2. Materials and Methods 

### 2.1. Plant Growth and Protoplast Assays

*Arabidopsis thaliana* wildtype and *tfiis* mutant (SALK_027258C) plants were grown in a growth chamber with an 8/16 h light/dark cycle at 22 °C. Both lines were obtained from Arabidopsis Biological Resource Center (Ohio State University, Columbus, OH, USA). Using 4-week old plants, protoplasts were isolated following an established protocol [49,50]. Briefly, we included 3% cellulose (Onozuka Yakult Pharmaceutical IND., Tokyo, Japan) and 0.8% macerase (MilliporeSigma, Burlington, MA, USA) in the digestion buffer (0.4 M mannitol, 20 mM KCl, 20 mM MES, 10 mM CaCl_2_, 0.1% BSA, 5 mM β-mercaptoethanol, pH 5.7) for digesting leaf blades with the lower epidermis layer removed by tapes. After 1 h digestion, protoplasts were pelleted by centrifugation at 150X *g* for 1 min. The pelleted protoplasts were then incubated in the W5 buffer (5 mM MES, 154 mM NaCl, 125 mM CaCl_2_, 5 mM KCl, pH 5.7) on ice for 30 min followed by incubation for 10 min with the MMg solution (4 mM MES, 0.4 M mannitol, 15 mM MgCl_2_, pH 5.7) at room temperature. About 10^5^ cells in 200 µL MMg buffer were supplemented with 5 µg PSTVd^RZ-Int^ RNA [51,52], then incubated with 200 µL PEG solution (4 g PEG4000, 3.5 mL ddH_2_O, 2 mL 1 M mannitol, 1 mL 1 M CaCl_2_) for 5 min at room temperature. Finally, protoplasts were briefly washed with the W5 solution and then incubated in the WI solution (0.4 M mannitol, 20 mM KCl, 20 mM MES, pH 5.7) for 2 days at room temperature before RNA purification. The protoplast assay was repeated three times.

We extracted genomic DNA, using a published protocol [53] for genotyping using TF2Sf (5′-CGGTTGTAAAGAGGCTAAGGTGAA-3′) and TF2Sr (5′- CAACAGAACTTCCAGTGGTTGTCAC-3′) primer pair, and TF2Sr and LBb1 (5′-GCGTGGACCGCTTGCTGCAACT-3′) primer pair to detect wildtype cDNA and the T-DNA insertion, respectively.

### 2.2. Recombinant Protein Purification 

Recombinant TFIIIA-7ZF protein with an intein-chitin binding tag was expressed and purified following the method described previously [40] with few modifications. *E. coli* BL21 (DE3) Rossetta strain (MilliporeSigma, Burlington, MA, USA) were grown to A_600_ = 0.4–0.6. TFIIIA-7ZF protein was then induced by adding IPTG (final concentration 0.4 mM) and ZnCl_2_ (final concentration 5 µM) to the culture followed by shaking at 20 °C for 16 h. Cells were harvested by centrifugation at 6000X *g* for 20 min. Harvested cells were resuspended in the lysis buffer (20 mM Tris-HCl pH 8.5, 500 mM NaCl, 1 mM PMSF, 5 µM ZnCl_2_ and 0.1% (*v*/*v*) Triton X-100). Cells were lysed by sonicating with the Bioruptor (Diagenode, Denville, NJ, USA) followed by centrifugation at 15000× *g* for 20 min at 4 °C. Supernatant was collected and incubated for 1 h with chitin resin (New England Biolabs, Ipswich, MA, USA) that was pre-equilibrated with the lysis buffer. After incubation, chitin resin was washed with 10 bed volume of lysis buffer to remove non-specifically bound proteins followed by incubation for 18 h at room temperature in the cleavage buffer (10 mM Tris-HCl pH 7.5, 20 mM KCl, 5 µM ZnCl_2_ and 10 mM DTT) for on-column cleavage to release tagless TFIIIA-7ZF. Fractions containing tagless proteins were desalted using Sephadex-G25 (GE Healthcare Life Sciences, Pittsburgh, PA, USA) equilibrated with storage buffer (10 mM Tris-HCl pH 7.5, 20 mM KCl, 5 µM ZnCl_2_). The desalted protein was concentrated with an Amicon protein concentrator (MilliporeSigma, Burlington, MA, USA). Glycerol was then added to the final concentration of 20% (*v*/*v*) before storing at −20 °C.

### 2.3. Purification of Pol II from Wheat Germ

Purification of Pol II from wheat germ was carried out following a published protocol [54] with modifications. All operations were performed at 4 °C, and all centrifugations were carried out for 15 min. One hundred grams wheat germ (Bob’s Red Mill, Milwaukie, OR, USA) was ground in a Waring Blender with 400 mL of buffer A (50 mM Tris-HCl pH 7.9, 0.1 mM EDTA, 1 mM DTT, and 75 mM (NH_4_)_2_SO_4_). The resulting homogenate was diluted with 100 mL of buffer A and followed by centrifugation at 15000× *g*. Supernatant was filtered through one layer of Miracloth (MilliporeSigma, Burlington, MA, USA). The resulting crude extract containing Pol II was precipitated by an addition of 0.075 volume of 10% (*v*/*v*) Polymin P with rapid stirring. The resulting mixture was subject to centrifugation at 10000× *g*. The pellet was washed with 200 mL of buffer A. The insoluble fraction, which contains Pol II, was resuspended with the buffer B (50 mM Tris-HCl pH 7.9, 0.1 mM EDTA, 1 mM DTT, and 0.2 M (NH_4_)_2_SO_4_). The resulting suspension was centrifuged at 10000X *g* to remove insoluble pellets.

(NH_4_)_2_SO_4_ precipitation was carried out by slowly adding 20 g of solid (NH_4_)_2_SO_4_ per 100 mL of the above supernatant with stirring. Mixture was centrifuged, and the pellet was dissolved in buffer C (0.05 M Tris-HCl pH 7.9, 0.1 mM EDTA, 1 mM DTT, 25% ethylene glycol) plus 0.1% Brij 35 (Thermo Fisher Scientific, Waltham, MA, USA) to make final (NH_4_)_2_SO_4_ concentration 0.15 M. The (NH_4_)_2_SO_4_ concentration was determined by conductivity. The resulting solution was applied to DEAE Sepharose FF (GE Healthcare Life Sciences, Pittsburgh, PA, USA) equilibrated with buffer C plus 0.15 M (NH_4_)_2_SO_4_. Then column was washed with five bed volume with buffer C containing 0.15 M (NH_4_)_2_SO_4_. Finally, bound Pol II was eluted with buffer C containing 0.25 M (NH_4_)_2_SO_4_. Fractions containing Pol II were pooled. The (NH_4_)_2_SO_4_ concentration was adjusted to 75 mM by conductivity. Resulting solution was applied to SP Sepharose FF (GE Healthcare Life Sciences, Pittsburgh, PA, USA) equilibrated with the buffer C containing 75 mM (NH_4_)_2_SO_4_. After washing the column with the same buffer, Pol II was eluted using the buffer C containing 0.15 M (NH_4_)_2_SO_4_. Eluted fractions containing Pol II were pooled. Ethylene glycol (VWR Chemicals BDH, Radnor, PA, USA) was added to a final concentration of 50% (*v*/*v*) before storing at −20 °C. 

### 2.4. Immunoblotting

The purified protein samples together with the BLUItra prestained protein ladder (FroggaBio, Wheatfield, NY, USA) were separated on an SDS-PAGE gel, followed by transferring to an Amersham Protran 0.45 NC nitrocellulose membrane (GE Healthcare Lifesciences, Pittsburgh, PA, USA) using the Mini-PROTEAN Tetra Cell (BioRad, Hercules, CA, USA). After 1 h incubation with 1% (*w/v*) nonfat milk in 1X TBS (50 mM Tris-HCl, pH 7.5, 150 mM NaCl) at room temperature, the 8WG16 monoclonal antibody (Thermo Fisher Scientific, Waltham, MA, USA) at 1:1000 against the largest subunit of Pol II was added and incubated overnight at 4 °C. After three washes with 1X TBST (50 mM Tris-HCl, pH 7.5, 150 mM NaCl, 0.1% Tween 20), HRP-conjugated secondary antibody against mouse IgG (Millipore Sigma, Burlington, MA, USA) was added at 1:8000 dilution. Membrane was washed three times with 1X TBST and incubated with HRP substrates (Li-COR Biosciences, Lincoln, NE, USA). The signals were detected with C-DiGit (Li-COR Biosciences, Lincoln, NE, USA).

### 2.5. RNA Preparation In Vitro

PSTVd RNA circularization was performed following a previously described method [55]. Ten µg of linear PSTVd RNA was incubated at 37 °C for 2 h in a final volume of 200 µL containing 150 units of calf intestinal alkaline phosphatase (New England Biolabs, Ipswich, MA, USA), 1X calf intestinal alkaline phosphatase buffer, and 80 units of RNase inhibitor (New England Biolabs, Ipswich, MA, USA). After incubation, the phenol-chloroform-based method was used to remove proteins followed by precipitation using ethanol. RNA pellet was resuspended in 10 µL nuclease-free water and incubated at 37 °C for 2 h in a final volume of 100 µL containing 100 units of T4 polynucleotide kinase (New England Biolabs, Ipswich, MA, USA), 80 units of RNase inhibitor (Thermo Fisher Scientific, Waltham, MA, USA), 1X T4 polynucleotide kinase buffer, and 1 mM ATP. Following incubation, proteins were removed using the phenol/chloroform method, followed by precipitation using ethanol. The pellet was dissolved in 20 µL of nuclease free water and incubated at 37 °C for 2 h in a final volume of 200 µL containing 1 mM ATP, 150 units of T4 RNA ligase (New England Biolabs, Ipswich, MA, USA), 80 units of RNase inhibitor, and 1X T4 RNA ligase buffer. Circular PSTVd RNA was separated by a 5% (*w/v*) polyacrylamide/8 M urea gel and detected by the Gel Doc XR+ gel documentation system (BioRad, Hercules, CA, USA) after ethidium bromide staining before gel excision. The excised gel was incubated with 0.3 M NaCl at 4 °C overnight. Gel pieces were removed using Costar Spin-X column (Corning, Corning, NY, USA) by centrifugation. The circular PSTVd RNA in the flow-through fraction was precipitated using ethanol. The resulting pellet containing circular RNA was resuspended in nuclease-free water and stored in −80 °C.

To prepare digoxigenin (DIG)-labeled (+)-strand and (−)-strand probes for RNA gel blots, *Sma*I-linearized pInt^95-94^(+) and pInt^95-94^ (−) were used as templates for in vitro transcription with T3 MAXIscript (Thermo Fisher Scientific, Waltham, MA, USA). For detecting 5S rRNA, (DIG)-labeled riboprobes were transcribed from a *Nco*I-linearized pGEMT-5SrRNA template [40] using a SP6 MAXIscript kit (Thermo Fisher Scientific, Waltham, MA, USA).

To prepare in vitro transcripts for the protoplast transfection assay, PSTVd^RZ-Int^ plasmid [51,52,56] was linearized by *Hind*III (New England Biolabs, Ipswich, MA, USA). The PSTVd^RZ-Int^ construct in pTZ18R was originally described by Hu et al. [52], and the 5′-Hammerhead ribozyme-PSTVd-Paperclip ribozyme-3′ cassette was later cloned into pGEM-T [51,56]. The linearized plasmid was used as the template for in vitro transcription using T7 MEGAscript (Thermo Fisher Scientific, Waltham, MA, USA) following the manufacturer’s manual. The products were purified using the MEGAclear kit (Thermo Fisher Scientific, Waltham, MA, USA). The products contain four RNA species, including the unit-length (+)-PSTVd as shown in Jiang et al. [50]. To prepare the size markers, the PSTVd^RZ-Int^ plasmids [51,52] were digested with *Hind*III (New England Biolabs, Ipswich, MA, USA) and then were subjected to in vitro transcription using T7 MEGAscript (Thermo Fisher Scientific, Waltham, MA, USA).

### 2.6. Pol II-catalyzed In Vitro Transcription

Pol II-catalyzed in vitro transcription was carried out using a previously developed protocol [7,22,40]. For DNA-dependent RNA transcription, 50 µL reaction contained 50 mM HEPES-KOH pH 7.9, 2 mM MnCl_2_, 100 mM KCl, 10% (*v*/*v*) glycerol, 1 U/µL SuperaseIn RNase inhibitor (Thermo Fisher Scientific, Waltham, MA, USA), 0.5 mM rATP, 0.5 mM rCTP, 0.5 mM rGTP, 0.35 mM rUTP, 0.15 mM DIG-labeled (Enzo Life Sciences, Farmingdale, NY, USA) rUTP, 150 ng DNA template, and 300 ng of partially purified Pol II. For RNA-dependent RNA transcription, 4 µM BSA (New England Biolabs, Ipswich, MA, USA) and TFIIIA-7ZF were treated with 1 unit of Turbo DNase (Thermo Fisher Scientific, Waltham, MA, USA) for 10 min at 37 °C. Then, 360 ng circular PSTVd, 300 ng partially purified Pol II, pretreated BSA and various amounts of TFIIIA-7ZF were incubated at 28 °C for 15 min before adding reaction buffer containing 50 mM HEPES-KOH pH 7.9, 1 mM MnCl_2_, 6 mM MgCl_2_, 40 mM (NH_4_)_2_SO_4_, 10% (*v*/*v*) glycerol, 1 unit/µL SuperaseIn RNase inhibitor (Thermo Fisher Scientific, Waltham, MA, USA), 0.5 mM rATP, 0.5 mM rCTP, 0.5 mM rGTP, 0.5 mM rUTP. Transcription reactions were incubated at 28 °C for 4 h. The reactions were terminated by 0.8 U/µL proteinase K (New England Biolabs, Ipswich, MA, USA) treatment at 37 °C for 15 min, followed by incubation at 95 °C for 5 min. The Pol II-catalyzed in vitro transcription assay was repeated three times.

### 2.7. RNA Purification. RNA Gel Blots, and Dot Blots

Transfected protoplasts were subjected to centrifugation at 1000× *g* for 2 min. The solution was removed, followed by wash with 1X PBS once. The pellet protoplasts were mixed with 100 µL RNAzol RT (Molecular Research Center, Cincinnati, OH, USA) and 35 µL ddH_2_O. RNA was purified following the manufacturer’s manual. For dot blots, DNA-dependent reactions were treated with Turbo DNase (Thermo Fisher Scientific, Waltham, MA, USA) for 20 min and then subjected to RNA purification by mixing 2 volumes ethanol followed by 14000× *g* centrifugation at 4 °C. The precipitated RNA was dissolved in 10 µL nuclease-fee H_2_O.

Total RNA from protoplasts or RNA from Pol II-catalyzed in vitro transcription were separated on a 5% (*w/v*) polyacrylamide/8 M urea gel for 1 h at 200 V. Then RNA was transferred to Hybond-XL nylon membrane (GE Healthcare Lifesciences, Pittsburgh, PA, USA) using the Trans-Blot SD semi-dry transfer cell (BioRad, Hercules, CA, USA) followed by UV cross-linking. Membranes were blocked by ULTRAhyb ultrasensitive hybridization buffer (Thermo Fisher Scientific, Waltham, MA, USA) followed by overnight hybridization with (DIG)-labeled riboprobes at 65 °C. Following the instructions of the DIG northern starter kit (MilliporeSigma, Burlington, MA, USA), membranes were washed and incubated with antibody against DIG label. Transcripts were identified using the Immun-Star AP chemiluminescence kit (BioRad, Hercules, CA, USA). Signals were obtained using C-DiGit (Li-COR Biosciences, Lincoln, NE, USA). Transcription products of DNA-dependent reactions were spotted on a Hybond-XL nylon membrane (GE Healthcare Lifesciences, Pittsburgh, PA, USA) followed by UV cross-linking. Membranes were blocked with ULTRAhyb ultrasensitive hybridization buffer (Thermo Fisher Scientific, Waltham, MA, USA), washed and incubated with the specific antibody against DIG labeling (MilliporeSigma, Burlington, MA, USA). The signals were detected with C-DiGit (Li-COR Biosciences, Lincoln, NE, USA). 

## 3. Results

### 3.1. An Efficient Method to Partially Purify Functional Pol II

**Figure 1:**
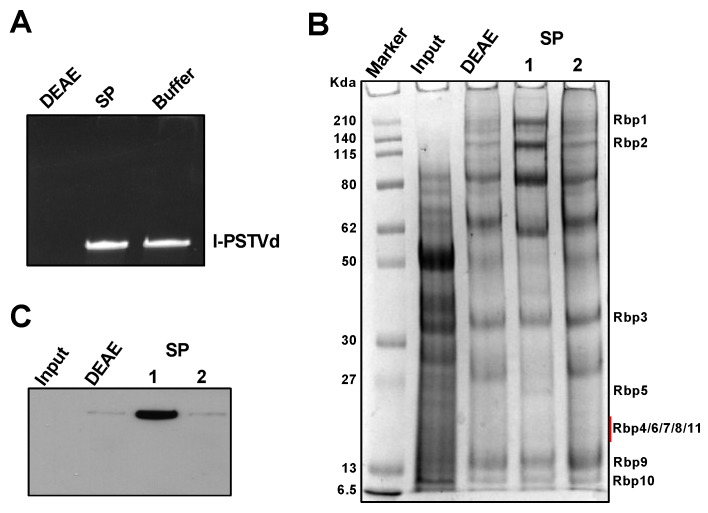
Pol II Purification. (**A**) Elution from the DEAE Sepharose FF column contains nuclease activity as demonstrated by RNA degradation in lane DEAE. The elution from DEAE was further purified using the SP Sepharose FF column to remove the nuclease activity, as shown in the SP lane. l-PSTVd, 480 ng linear PSTVd; (**B**) Purified protein factions were visualized in an SDS-PAGE gel after coomassie blue staining. Protein bands corresponding to the putative Pol II subunits were labeled based on the molecular weight of the corresponding homologs in *Arabidopsis*. See Table S1 for details; (**C**) Immunoblots detection of the largest subunit of Pol II, Rbp1, using the 8WG16 antibody. Input, raw lysate of wheat germ. DEAE, the elution fraction from the DEAE Sepharose FF column. SP, the elution fractions (1 and 2, elution using 0.15 M and 0.3 M ammonium sulfate, respectively) from the SP Sepharose FF column. Buffer, protein storage buffer.

We recently established a Pol-II-catalyzed in vitro system to study RNA-templated transcription [40]. In this system, we mixed circular PSTVd as the RNA template, recombinant TFIIIA-7ZF protein purified from *E. coli*, and immuno-purified Pol II from plants. To obtain immune-purified Pol II complex, we agroinfiltrated *Nicotiana benthamiana* plants with a plasmid harboring a FLAG-tag fused cDNA of the second largest subunit of Pol II cloned from *A. thaliana* [57]. However, the Pol II expressing efficiency varied among batches. To circumvent this shortcoming, we revisited a Polymin P (PEI)-based purification method that was developed in the mid-1970s to prepare active Pol II from wheat germs [54]. At low ionic strength, PEI precipitates DNA genomes and many DNA-binding proteins. Following re-suspension, the protein solution passes through the DEAE Sepharose FF column and the phosphocellulose column sequentially. The final elution contains the major components of the Pol II complex. However, the phosphocellulose column has been discontinued. Using the DEAE Sepharose FF column alone resulted in the contamination of strong nuclease activities in the purified Pol II fraction (Figure 1A). After some initial trials, we found that the SP-Sepharose column can replace the phosphocellulose column to remove nucleases (Figure 1A) and obtain the relatively pure Pol II complex (Figure 1B). Using the specific antibody against the largest subunit of Pol II, we confirmed that the purified fraction contains Pol II (Figure 1C). 

**Figure 2:**
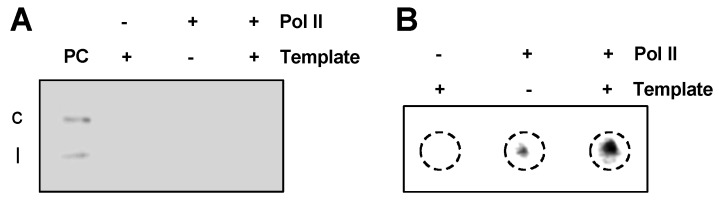
The activity of purified Pol II on DNA templates. The purified Pol II cannot generate specific RNA products from the p35S::RZ-PSTVd plasmid (**A**) but did possess nucleotide incorporating activity as shown in a dot blot (**B**). PC, the mixture of circular and linear PSTVd RNA served as the positive control. c, circular PSTVd. l, linear PSTVd.

We then tested the activity of purified Pol II for transcription. When we used p35S::RZ-PSTVd plasmid [51] as the template, we could not observe any specific band in RNA gel blots using the PSTVd specific probe (Figure 2A). This is expected because the purified polymerase fraction likely lacks the necessary general transcription factors to initiate DNA-directed transcription from specific promoters [58,59]. To confirm that the purified Pol II possesses the enzymatic activity, we performed dot blots to examine the rNTP incorporation efficiency. Using the same DNA template, we supplied the rNTPs mixed with (DIG)-labeled rUTP for Pol II to perform transcription. The products were precipitated to remove free DIG-rUTP before dot blots. As shown in Figure 2B, dot blots showed that the purified Pol II was active for rNTP incorporation, evidenced by a much stronger signal. The signal in the middle of Figure 2B might be attributable to the precipitated Pol II with incorporated (DIG)-labeled rUTP nucleotides. Nevertheless, the signal in the reaction without DNA templates is much weaker than that from the reaction with the supply of DNA templates. 

### 3.2. Circular Templates are Critical for Generating Longer-Than-Unit-Length Intermediates

**Figure 3:**
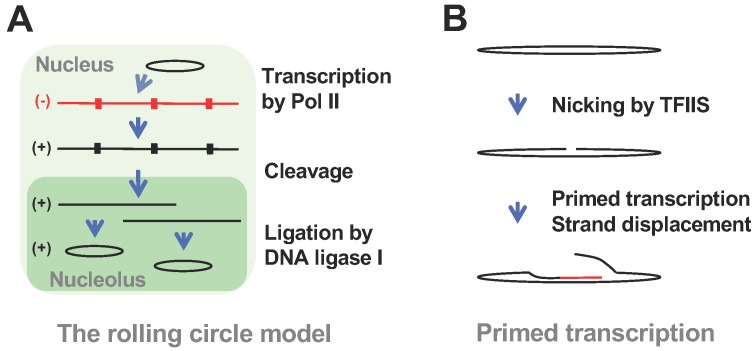
PSTVd rolling circle model (**A**) and a hypothetical primed transcription scheme (**B**). The nodes in the (−)- and (+)-concatemers in (**A**) depict the cleavage sites for generating unit-length copies. Black lines depict sense orientation of PSTVd, while red lines depict antisense orientation of PSTVd.

The discovery of the circular (+)-PSTVd [60,61], concatemeric (−)-PSTVd [46], and the double-stranded RNA complex containing the unit-length and the longer-than-unit-length (+)- and (−)-PSTVd [62,63] led to the proposal of an asymmetric rolling circle model of PSTVd replication [47,48] (Figure 3A). It has been assumed that Pol II catalyzes the transcription de novo using PSTVd circular RNA templates to generate multimeric (−)-PSTVd intermediates. In contrast, if Pol II employs the TFIIS-based primed transcription mechanism for PSTVd, it would generate the chimeric (+)-PSTVd and (−)-PSTVd fusion intermediates with a length ranging from close to the unit-length to oligomeric PSTVd pending on the nick site (Figure 3B). In the primed transcription scenario, a circular RNA template may not be required, since the nicked 3′-end of the linear PSTVd can serve as a primer.

**Figure 4:**
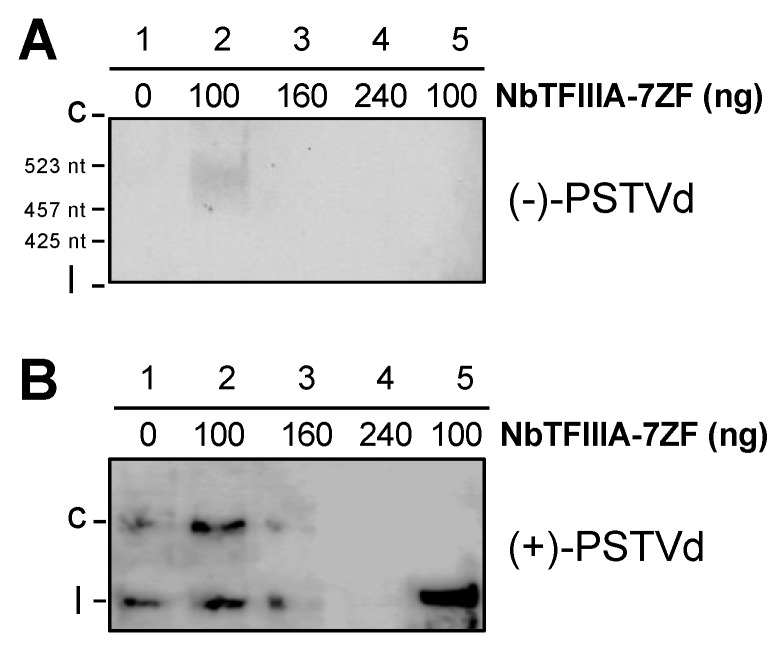
Pol II-catalyzed transcription using PSTVd circular RNA templates. (**A**) In vitro transcription products detected by the (+)-PSTVd riboprobe; (**B**) The PSTVd templates detected by the (−)-PSTVd riboprobe. Lanes 1–4 were supplied with circular PSTVd as templates, while lane 5 was supplied with linear PSTVd as the template. c, circular PSTVd. l, linear PSTVd.

Using the partially purified Pol II from wheat germ, we performed the in vitro transcription assay and test the activity of the purified Pol II in catalyzing transcription on RNA templates. Half of the reaction mixture was subjected to RNA gel blots to detect products (Figure 4A), while the other half was used to detect templates (Figure 4B). As shown in Figure 4A, the purified Pol II alone exhibits very weak activity in transcribing PSTVd circular RNA templates. Adding 100 ng TFIIIA-7ZF to the in vitro transcription significantly enhanced the Pol II activity on the circular RNA template and led to the production of the longer-than-unit-length (−)-strand products. Interestingly, compared with the supplementation of 100 ng TFIIIA-7ZF, adding more TFIIIA-7ZF led to the drastic reduction of circular templates and failure in generating the longer-than-unit-length (−)-strand product (Figure 4). Occasionally, we observed the circular template remained at a comparable concentration with 100 ng and 160 ng TFIIIA-7ZF in the reactions but still only detected the longer-than-unit-length (−)-strand products in the reaction with 100 ng TFIIIA-7ZF (Figure S1). These observations demonstrated that the ratio of TFIIIA-7ZF and the circular template is critical for generating (−)-PSTVd intermediates. Under the condition for efficient synthesis of (−)-PSTVd intermediates, the stoichiometric ratio among TFIIIA-7ZF, Pol II, and the PSTVd circular template is estimated to be 2:1:1 based on protein and RNA concentrations. The template degradation is likely attributable to the presence of divalent ions (i.e., Mn^2+^) in the reaction, because incubating the circular templates with Mn^2+^ alone also resulted in a significant reduction in circular templates (Figure S2). It is noteworthy that a mixture of 100 ng TFIIIA and 300 ng Pol II, the condition allowing effective transcription on circular RNA templates, failed to generate longer-than-unit-length product using the linear (+)-PSTVd template (Figure 4). Given the importance of the circular template, the proposed HDV primed transcription [36,37,38,39], using the activity of the TFIIS/Pol II complex to nick the circular RNA and free the cleaved 3′end as the primer, unlikely exist in PSTVd replication. 

### 3.3. PSTVd Replication is Independent of TFIIS

Since TFIIS plays a crucial role in the proposed primed transcription model for HDV [36], we decided to test its role in PSTVd replication using the *tfiis* loss-of-function *Arabidopsis* mutant for the protoplast transfection assay. TFIIS is critical for plant growth [64,65]. Albeit the homozygous *tfiis* loss-of-function *Arabidopsis* mutant is viable [64], loss of TFIIS results in early germination [64] and reduced tolerance to UV radiation [66]. In addition, more than seven hundred genes encoded in the nuclear genome exhibited significant differential expression patterns in the *tfiis* mutant [64]. 

**Figure 5:**
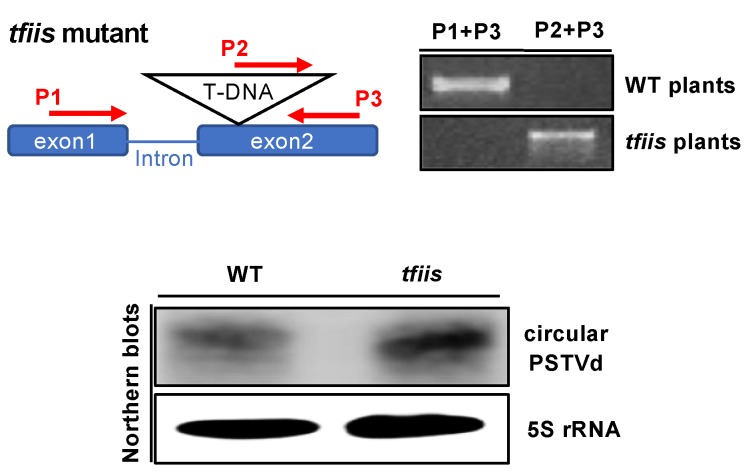
PSTVd replication in protoplasts. Top panel shows the schematic presentation of T-DNA disruption in the TFIIS locus and the genotyping result. The genotyping PCR, using two sets of primers (P1+P3 and P2+P3) confirmed wildtype plants and *tfiis* mutants. The bottom panel shows PSTVd replication in wildtype and *tfiis* protoplasts. The sequence of P2 primer is derived from the inserted T-DNA, while the P1 and P3 primers are derived from the endogenous gene. WT, wildtype.

We chose SALK_027258C as the material because it is a well-established *tfiis* loss-of-function mutant [64]. As shown in Figure 5, genomic PCR confirmed that our protoplasts were generated from homozygous *tfiis* loss-of-function plants. Using a previously established protoplast transfection system [50], we transfected wildtype and mutant protoplasts with linear PSTVd RNA inoculum and harvested the total RNA two days post inoculation. As shown in Figure 5, we could observe the accumulation of circular PSTVd in both wildtype and *tfiis* protoplasts, which reflects the successful replication of PSTVd based on previous studies [45,50,56,67,68]. Thus, our observation indicates that PSTVd replication is unlikely via the TFIIS-based primed transcription mechanism.

## 4. Discussion

The RdRP activity of Pol II is critical for gene regulation [33,34] and pathogen replication (i.e., HDV and nuclear-replicating viroids) [16,69,70]. Pol II initiates transcription de novo on DNA templates in all eukaryotic cells [35] but utilizes the primed transcription mechanism on B2 noncoding RNA templates in mammalian cells [34]. Structural analysis [36] and some replication assays [36,37,38,39] also suggested that Pol II may utilize the primed transcription mechanism for HDV infection, with the aid of TFIIS activity [36]. Whether the primed transcription mechanism is common for Pol II to transcribe RNA templates deserves to be clarified. 

Nuclear-replicating viroids rely on Pol II activity for replication [18,19,20,21,22], which has long been assumed to be de novo transcription. The discoveries of circular PSTVd [60,61], longer-than-unit-length (−)-PSTVd [46], as well as the double-stranded RNA composed of unit-length and longer-than-unit-length (+)- and (−)-PSTVd [62,63] led to the proposal of the rolling circle replication model [47]. Later, some evidence denied the existence of circular (−)-PSTVd and established the asymmetric rolling circle mechanism [48]. The asymmetric rolling circle replication is also common to all nuclear-replicating viroids [69,71]. 

Here, we collected experimental evidence directly showing that circular (+)-PSTVd, but not the unit-length linear (+)-PSTVd, serves as the template for Pol II-catalyzed transcription. The generation of longer-than-unit-length (-)-strand intermediates depends on the concentration of TFIIIA-7ZF and circular (+)-strand templates. When using the circular (+)-PSTVd as the template in our in vitro transcription assay, the circular RNA is often degraded to generate unit-length linear (+)-PSTVd and smaller fragments (Figure 4B). The longer-than-unit-length products could not be detected when the circular templates were completely degraded (Figure 4A). In addition, linear (+)-PSTVd RNA alone failed to serve as templates for generating longer-than-unit-length product (Figure 4), indicating that only the circular (+)-PSTVd serves as the template for the synthesis longer-than-unit-length (−)-PSTVd intermediates catalyzed by Pol II. Furthermore, we presented direct evidence that TFIIS is not required for PSTVd replication in protoplast cells (Figure 5). These observations, together, strongly support that Pol II catalyzes de novo transcription using PSTVd circular RNA templates.

It is also noteworthy that our purified Pol II cannot enable promoter-based transcription on DNA for the synthesis of specific products (Figure 2A), which is in line with previous reports that the Pol II enzyme requires a cohort of factors to enable regulated transcription [58,59]. However, this purified Pol II is able to utilize circular PSTVd RNA templates to synthesize the longer-than-unit-length products, with the aid of TFIIIA-7ZF in a dosage-dependent fashion (Figure 4A). This observation further confirmed the importance of TFIIIA-7ZF in RNA-templated transcription catalyzed by Pol II, particularly for the regulation of Pol II processivity. Given that TFIIIA-7ZF is a novel transcription factor for RNA-templated transcription and TFIIS, as a conserved general transcription factor, is dispensable for the PSTVd RNA-templated transcription catalyzed by Pol II, this polymerase should employ a novel machinery to catalyze transcription on PSTVd RNA templates. This finding has significant implications in other RNA-templated transcription by DdRPs.

## Supplementary Materials

The following are available online at https://www.mdpi.com/1999-4915/12/4/371/s1, Figure S1: Pol II-catalyzed transcription depending on the TFIIIA-7ZF/template ratio. (A) In vitro transcription products detected by the (+)-PSTVd riboprobe; (B) The PSTVd templates detected by the (-)-PSTVd riboprobe. c, circular PSTVd. l, linear PSTVd. Figure S2: Templates degradation by reaction buffer component. c, circular PSTVd. l, linear PSTVd. Table S1: Pol II subunits. Based on reference 7.
