# Supplementary material for: Evidence Supporting That RNA Polymerase II Catalyzes De Novo Transcription Using Potato Spindle Tuber Viroid Circular RNA Templates
Source: Viruses. 2020 Mar 27;12(4):371. doi: 10.3390/v12040371 (PMC7232335; doi:10.3390/v12040371)
Supplement: Supplementary file 1 [file viruses-12-00371-s001.zip › viruses-737725-for conversion-suppl/Supplemental files/Figure S1.pptx]

## Slide 1
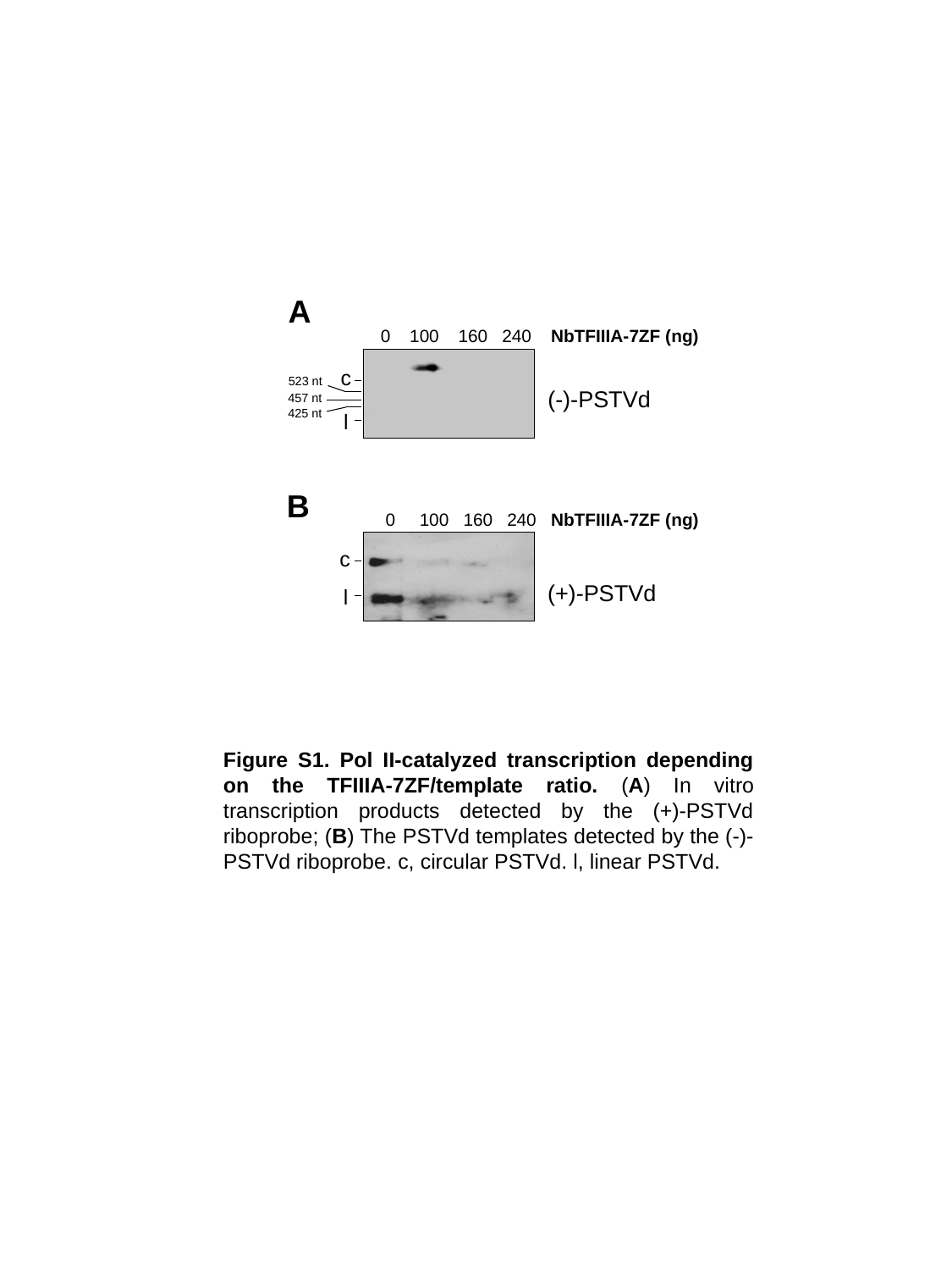

A
0 100 160 240 NbTFIIIA-7ZF (ng)
c
523 nt
(-)-PSTVd
457 nt
425 nt
l
B
0 100 160 240 NbTFIIIA-7ZF (ng)
c
(+)-PSTVd
l
Figure S1. Pol II-catalyzed transcription depending on the TFIIIA-7ZF/template ratio. (A) In vitro transcription products detected by the (+)-PSTVd riboprobe; (B) The PSTVd templates detected by the (-)-PSTVd riboprobe. c, circular PSTVd. l, linear PSTVd.
