# Supplementary material for: Evidence Supporting That RNA Polymerase II Catalyzes De Novo Transcription Using Potato Spindle Tuber Viroid Circular RNA Templates
Source: Viruses. 2020 Mar 27;12(4):371. doi: 10.3390/v12040371 (PMC7232335; doi:10.3390/v12040371)
Supplement: Supplementary file 1 [file viruses-12-00371-s001.zip › viruses-737725-for conversion-suppl/Supplemental files/Figure S2.pptx]

## Slide 1
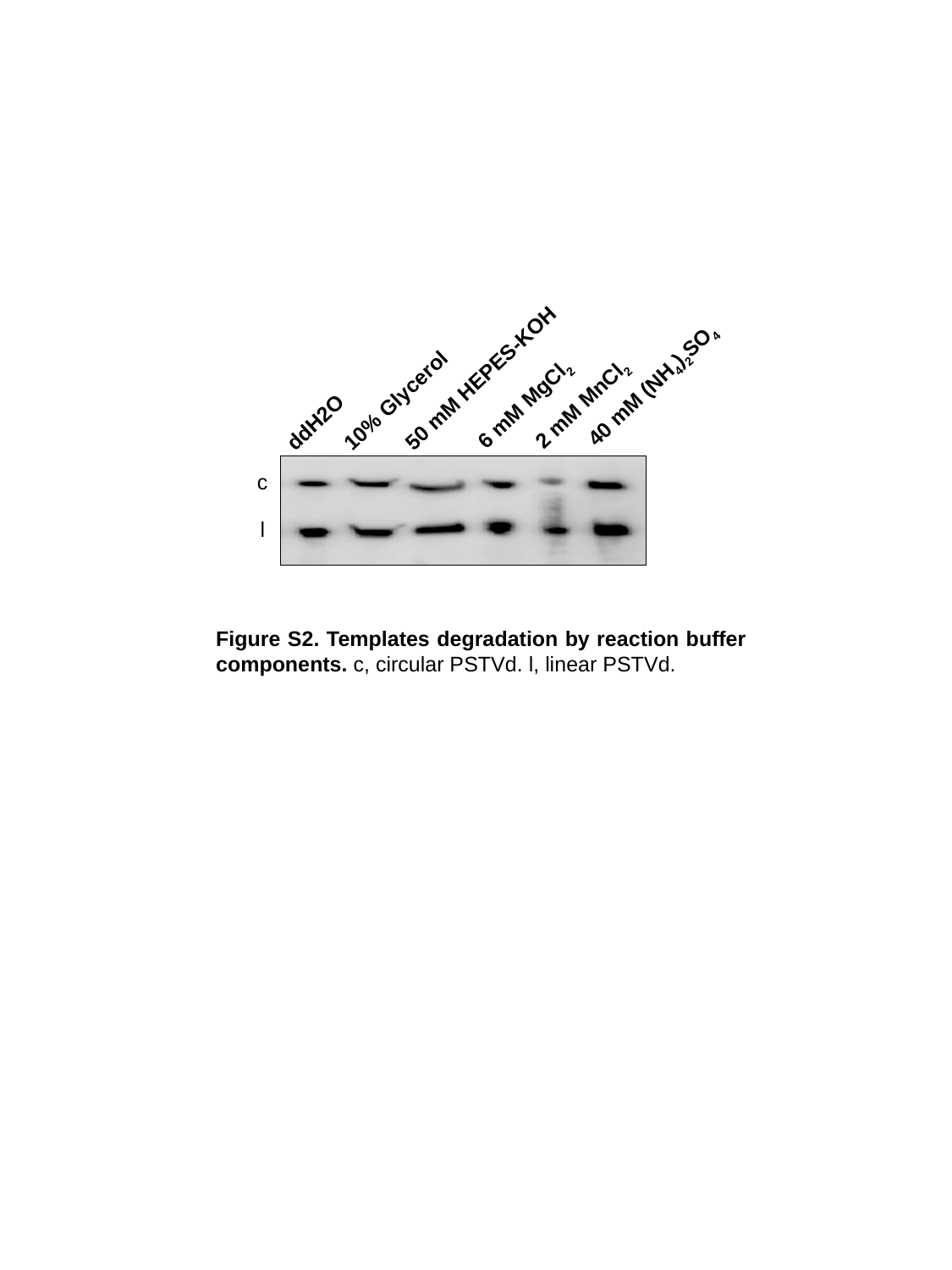

50 mM HEPES-KOH
40 mM (NH4)2SO4
10% Glycerol
6 mM MgCl2
2 mM MnCl2
ddH2O
c
l
Figure S2. Templates degradation by reaction buffer components. c, circular PSTVd. l, linear PSTVd.
